# Design and Implementation of an AI-Enabled Sensor for the Prediction of the Behaviour of Software Applications in Industrial Scenarios

**DOI:** 10.3390/s24041236

**Published:** 2024-02-15

**Authors:** Angel M. Gama Garcia, Jose M. Alcaraz Calero, Higinio Mora Mora, Qi Wang

**Affiliations:** 1 School of Computing, Engineering and Physical Sciences (CEPS), University of the West of Scotland (UWS), High Street, Paisley PA1 2BE, UK; angel.gama-garcia@uws.ac.uk (A.M.G.G.); qi.wang@uws.ac.uk (Q.W.); 2 Department of Computer Science and Technology, Universidad de Alicante, Carr. de San Vicente del Raspeig, 03690 Alicante, Spain; hmora@ua.es

**Keywords:** software application, virtualisation, AI-enabled sensor, prediction algorithm, random forest

## Abstract

In the era of Industry 4.0 and 5.0, a transformative wave of softwarisation has surged. This shift towards software-centric frameworks has been a cornerstone and has highlighted the need to comprehend software applications. This research introduces a novel agent-based architecture designed to sense and predict software application metrics in industrial scenarios using AI techniques. It comprises interconnected agents that aim to enhance operational insights and decision-making processes. The forecaster component uses a random forest regressor to predict known and aggregated metrics. Further analysis demonstrates overall robust predictive capabilities. Visual representations and an error analysis underscore the forecasting accuracy and limitations. This work establishes a foundational understanding and predictive architecture for software behaviours, charting a course for future advancements in decision-making components within evolving industrial landscapes.

## 1. Introduction

In the ever-evolving landscape of industrial operations in Industry 4.0 and 5.0, a clear shift towards virtual work has been seen [1]. The integration of virtualisation technologies in industry, such as those of cloud computing and mobile edge computing, is indispensable when adapting to the evolving demands of modern industrial landscapes. This leads to the next generation of distributed systems, where there is a clear symbiosis between hardware and software with impacts on key factors such as scalability, accessibility, and real-time data processing [2].

This softwarisation of industrial processes requires ambitious new approaches to address both hardware and software monitoring [3], which is driven by the critical need for real-time solutions. As a result, predictive maintenance systems (PDMs) have emerged as some of the most crucial strategies for adoption in modern industries [4]. These solutions aim to provide insights into system behaviour by using historical data [5,6], predicting behaviour [7], identifying anomalies, and facilitating comprehensive/immediate action.

Traditionally, industrial processes relied on dedicated hardware and software configurations for optimal performance and control. However, the functionality of software applications within the industrial framework is the key to the improvement of processes, resource utilisation, and, ultimately, successful outcomes. The monitoring and prediction of the behaviour of software applications in an industry present different challenges. Proactive maintenance strategies are capable of foreseeing how each running software works and anticipating potential issues [8] or even cybersecurity threats [9]. In this context, the effectiveness of software applications within a virtualised environment becomes pivotal for comprehending the overall system performance. The behaviour of software, including aspects such as CPU and memory usage, execution time, and disk reading/writing, plays a crucial role in determining a system’s operational health. Predicting these behaviours is critical for proactive maintenance strategies, as this enables the anticipation of emerging risks related to resource utilisation. Predictive models applied to these metrics directly contribute to the ability to identify and address potential issues.

Predictive monitoring is a field that is in continuous development, even more so with the growth of AI applications. This evolution is visible in various sectors and is exemplified in the case of aeronautical industry [10], where the use of machine learning for predictive maintenance has been introduced as a mandatory future step, or the wind energy industry [11] with the application of AI-driven predictors. Accurately predicting software behaviour is paramount for anticipating and, thus, proactively preventing potential issues, which leads to the minimization of business disruptions, reductions in losses, such as operational and financial losses, and ensuring uninterrupted digital services for end-users.

Some solutions, while valuable, may be limited in their application to the scope for which they were originally designed, or, in other words, they may adapt to the dynamic demands of modern softwarised industrial setups. This research aims to bridge the gap in resolving this industrial challenge. In addressing it, our approach encompasses the following contributions to the advancement of the state of the art:

- The design, implementation, and empirical validation of an architecture for gathering the metrics of distributed software applications (SAs).
- The design and implementation of software for performing the real-time compilation of a dataset of metrics. The validation was conducted by collecting 17 metrics, with more than 1000 measurements per metric, and more than 500 single threads of software applications.
- The design, implementation, and empirical validation of machine learning techniques for achieving real-time metric forecasting.

By applying these contributions, this study aimed to predict the behaviour of distributed software applications in the context of Industry 4.0 and 5.0. The focus was the development of a novel architecture that integrated the gathering of metrics and real-time forecasting by applying machine learning algorithms. This work is intended to contribute to the advancement of predictive maintenance strategies and operational insights, ultimately empowering autonomous decision making and self-optimisation processes in the dynamically evolving and increasingly demanding landscape of modern industrial setups.

The remainder of this manuscript is organised as follows. Section 2 reviews the current state of the art in the field of metric forecasting. Section 3 explains the proposed architecture for achieving the goal of an AI-enabled sensor for the prediction of the behaviour of industrial software applications. Then, Section 4 describes the implementation details and provides an end-to-end flow diagram of the proposed architecture from the gathering and reporting to the prediction. Section 5 introduces the dataset that was created. In Section 6, the prediction algorithm applied and the model used are explained. The empirical results are shown and analysed in Section 7. Finally, Section 8 discusses what has been achieved and future work prior to the conclusion of the paper in Section 9.

## 2. Related Work

In this section, a study of the related work on the prediction of metrics is carried out. It is focused on studies that employed machine learning algorithms and explores the methods and validation techniques used. By evaluating diverse approaches, it is intended to understand the current status of the literature in this field and, ultimately, comprehend the choices made in the work presented.

As we explore existing architectures in the domain of the prediction of metrics of software applications, it is essential to understand the diverse approaches employed in the literature. Selmy et al. presented a predictive framework in [12]. The architecture systematically collected data from sensors, employed a robust publishing/subscriber communication system, and conducted data preprocessing before executing advanced prediction algorithms such as ARIMA and LSTM; ultimately, the results were presented through visualisation. In [13], the authors presented work that also proposed a similar architecture. The datasets were built, features were selected, and machine learning algorithms were executed to predict code-smells. In general, it can be seen that various architectures share commonalities in terms of their modular components, which include agents for data gathering, communication middleware for seamless interactions, and predictors that utilise machine learning algorithms. However, there are also differences that are worth commenting on. The architecture presented here leveraged the use of different agents that separated the main tasks. A database was used to save and access data in real time or for future studies, together with an aggregator that provided high-level metrics, unlike in the previous literature, which focused on direct gathering, processing, and prediction.

In the search for accurate forecasting, various models were considered and evaluated before a choice was made. These models encompassed diverse methodologies. Random forest emerged as the preferred choice due to its ability to capture nonlinear relationships, unlike traditional regression models, such as ARIMA, which assume linear patterns. In addition, it uses multiple decision trees to mitigate the overfitting tendencies present in individual decision trees. Jain et al. conducted a comparative study of various models for the forecasting of load demand consumption in [14]. Their results showed that the random forest model achieved better performance than that of advanced deep learning models in four out of five test cases. They emphasised the effectiveness of simpler machine learning methods, cautioning against more complex and unneeded deep learning models. Another study [15] by Sedai et al. showed that the random forest model was the best-performing model among the four types of models tested (ARIMA, SVR, RF, and LSTM), with 50% better accuracy than that of univariate models and 10% better accuracy than that of multivariate models.

The JM1 dataset from the PROMISE Software Engineering Repository [16] is an example of a dataset used for software defect prediction. It is intended to identify modules that can have any type of defect. It is a collection of 10,885 modules from 14 different software systems. The dataset includes 22 metrics that characterise software modules, including lines of code, complexity measures, and Halstead metrics [17]. These metrics provide insights into the structure and complexity of the code, which can be correlated with the likelihood of defects. It is widely used, as can be seen in [18], where Rahim et al. proposed a Naïve Bayes Classifier for the identification of software application defects with a high accuracy, reaching 98.7%. The early detection of these defects in software systems can help developers remove them and, thus, improve the software quality before the deployment phase.

Regarding resource usage prediction, Sriram et al. presented a time-series forecasting method applied to the CPU percentage in [19]. They compared ARIMA and LSTM, and they concluded that the best results were obtained with a neural network model. Also, in [20], Wang et al. conducted a study of different neural networks to achieve high-accuracy prediction. In [21], a random forest autoscaler was applied to the prediction of the CPU and memory utilisation for containerised microservices. The authors created a dataset with the following features: CPU and memory usage, disk read bytes, disk write bytes, network in, and network out. Goli et al. presented a microservice autoscaler in [22]. They leveraged machine learning models to predict the end-to-end response latency in a microservice application. They used a large number of metrics, such as the CPU utilisation rate and network utilisation rate, and better results were achieved with random forest than with traditional regression algorithms.

Regarding I/O optimisation, Bagbaba [23] introduced a machine-learning-driven auto-tuning solution for improving collective I/O performance; the main goal was to predict the I/O performance based on the results of the previous runs.

The paramount importance of the prediction of metrics has been demonstrated in a variety of industries. Regarding the healthcare and medical industry, Chen et al. [24] presented a study that achieved blood pressure measurement prediction using a support vector machine regression model and a random forest regression model, and the best results were obtained with the latter. Shanmugarajeshwari and Ilayaraja [25] presented a system for efficiently mining the risk factors of chronic kidney disease. They achieved 98.97% accuracy with random forest, which was the algorithm with the best results and was the fastest among random forest, SVM, and an ANN. An interesting application of machine learning in a completely different industry can be found in [26], where Tian et al. applied different models, such as random forest, LSTM, WNN, and SVR, to the prediction of drought based on time-series imaging. It is of interest to see how random forest was used and compared to different methods, and, depending on the application, achieved better or worse results.

### Findings

The analysis of the related work revealed several limitations in existing approaches for the prediction of metrics. Table 1 shows a comparison based on criteria that allow us to understand differences between our approach and different contributions. Regarding the algorithm used, in several of the works that were mentioned, it was observed that random forest was one of the most commonly used algorithms for time-series regression forecasting. Model comparisons in which it provided successful results can be easily found.

Additionally, it was difficult to find a study that took as large of a number of metrics as in our work into account. The largest number of outputs (forecast metrics) was 2, as almost all of the studies had a single output (with the exception of [21]). Only the authors of [19,21,23,24] automated the gathering of the metrics and their further prediction, and the rest of the studies tested with static data. Only one was found to have a distributed architecture [19], and the validation of their work was mainly analytical or based on simulations. It was difficult to find a prototype that worked completely in real time and to find strong validations of the proposed methods, which was one of the main motivations of this research work.

Furthermore, regarding software applications, the works most aligned with ours were focused on specific resources or the scaling of microservices. This demonstrates a gap in the literature, that is, the limited comprehension and prediction capabilities concerning software applications. Addressing this gap serves as a motivation behind the approach introduced in our study.

## 3. Proposed Architecture

**Figure 1:**
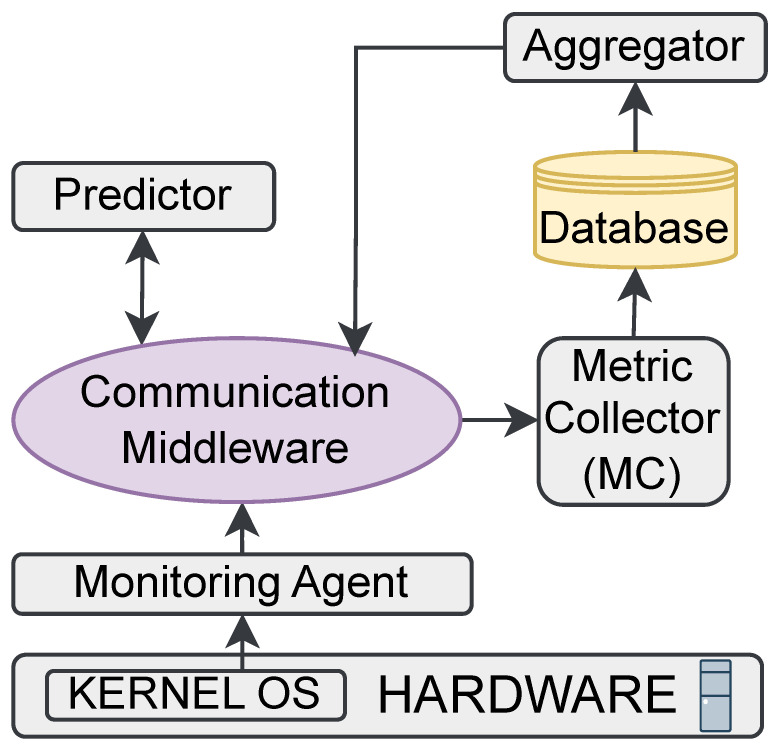
Proposed architecture for enabling the sensing and prediction of software application metrics.

In this section, the architecture that was designed to enable the sensing and prediction of software application metrics is presented. The architecture comprises a series of interconnected agents. The architecture collaborates with AI to achieve the contributions of the sensing and prediction of software application metrics in industrial scenarios. An overview of the architecture and the set of components is depicted in Figure 1. They are described in the following subsections.

### 3.1. Monitoring Agent (MA)

The monitoring agent is in charge of sensing all of the software applications running within a system. It achieves the profiling of the software behaviour by obtaining the metrics for each of the processes and the threads that form the software applications running in the system. The MA gathers metrics not only from the host system, but also from docker containers [27], allowing OS-level virtualisation, virtual machines, etc. It is fully configurable so that new metrics can be added as new rules with definitions and ways of retrieving them. In summary, the MA gathers vital software performance data and publishes them in the publication/subscription middleware. To maintain the separation of data, a consistent routing key convention is employed with the format “software.ma.IP”. In this convention, if the IP is, for instance, “192.168.121.2”, the routing key will be “software.ma.192.168.121.2”. This meticulous organisation of the publication topic ensures that each software metric is processed and stored regardless of the system to which it belongs.

### 3.2. Metric Collector (MC)

This component subscribes to channels in which different types of metrics—either raw measured metrics or predicted ones—are published. It receives them and securely stores their data in a dedicated database. This ensures data integrity and availability for future access by any other component of the architecture. This component subscribes to “software.ma.#”, meaning that it receives the metrics generated by all different MAs distributed throughout the system.

### 3.3. Aggregator

The aggregator is in charge of providing high-level metrics that are both spatial and temporal aggregations of raw metrics, as well as linear or nonlinear combinations of raw metrics. These are useful for providing objective KPIs or other high-level metrics. Temporal aggregation is performed by using any aggregator function, such as the median, average, max, or min. Spatial aggregation is implemented by using combinations of different metrics. The new metrics from the aggregator are treated in the same way as that of raw metrics and, thus, published to the same publishing channel. Thus, both raw metrics and aggregated metrics are treated similarly by the predictor.

### 3.4. Predictor

In the final processing stage, this component forecasts future values based on the metrics received from the middleware. It uses random forest to generate predictions for software application metrics within the system. Furthermore, those predictions are published in the middleware for its future use in other applications, providing valuable insights for future decision making and planning.

The prediction function within the architecture is formulated as described in the following.

#### Variables and Definitions

Let *H* represent a set of histograms in which each histogram $H_{i}$ is a collection of metric values for a given metric $M_{i}$ from time t=1 up to the current time $t_{\mathrm{now}}$:

$$H=\{H_{1},H_{2},\ldots ,H_{n}\}$$

$$H_{i}=\overset{t_{\mathrm{now}}}{\underset{t=1}{⋃}}M_{i}^{t}$$

$P_{i}$ represents the predictive function for metric $M_{i}$, denoted as $f(H_{i})$, which aggregates the histograms $H_{i}$ to derive the next value $M_{i(n+1)}$:

$$P_{i}=f\left(H_{i}\right)=M_{i(n+1)}$$

The predictive function (*f*) processes the combined histograms ($H_{i}$) and generates the next value $M_{i(n+1)}$.

### 3.5. Communication Middleware

The middleware serves for communication to link every agent of the architecture, and it plays a crucial role in facilitating their interaction. This component, while abstracted from the individual agents, is in charge of the orchestration of message queues and ensures seamless communication flow. By providing a channel for asynchronous data transmission, the middleware enables the efficient decoupling of the components, allowing each agent to independently perform its designated tasks while maintaining synchronisation and integrity in data processing.

## 4. Implementation of the Approach

It is important to understand the practical implementation and functioning of this architecture. This section dives into the implementation details of our approach. The specific technologies and tools used in each task are introduced. Moreover, the flow followed by the components is shown and described.

### 4.1. Implementation Details

All of the components of the architecture were prototyped in Java 13, with the exception of the predictor, for which python3 was used. Furthermore, the message broker in charge of the data communication was RabbitMQ version 3.12.7. It was based on the Advanced Message Queuing Protocol (AMQP). This protocol provides a standardised messaging format and ensures interoperability between different message brokers and client applications. It was chosen due to its high scalability and fault tolerance, which enabled us to handle large volumes of messages and ensured that message delivery was not affected by system failures. All data were stored and accessed through a relational database system, MySQL 8.1, which ensured data integrity and efficient retrieval.

**Figure 2:**
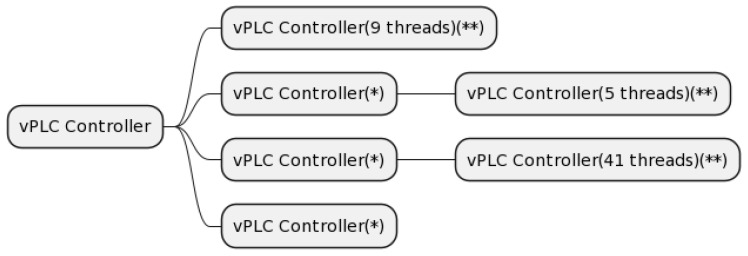
Software application example: “Virtual PLC Controller”. (*) Child of a primary process, (**) threads.

Regarding software applications, they were defined as computer programs designed to carry out a task for a specific purpose. They could be composed of a single process or set of processes/threads that worked as one to achieve a function. Figure 2 shows a process tree representation of the software application “virtual PLC Controller” in Linux OS (initialised as vPLC Controller). It was formed by a set of processes and threads. The primary process served as the root, and it had three child processes that spawned from it and nine threads. Those child processes were, at the same time, parent processes of several threads. This structure is common in complex systems in which an application needs more than one parallel task to be performed. In this case, the controller could have three child processes executing parallel jobs, such as continuously receiving data from a sensor while processing them and communicating with different actuators.

### 4.2. Sequence Diagram

**Figure 3:**
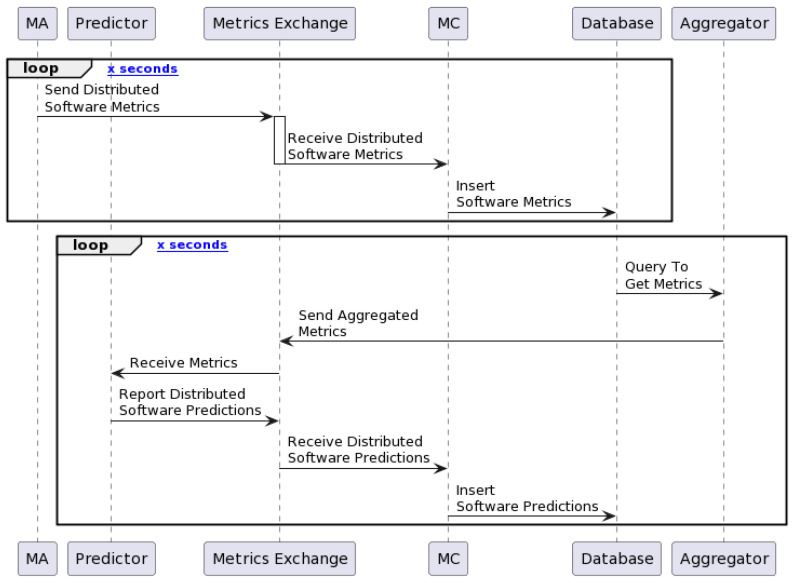
Workflow of the proposed architecture.

The flow depicted in the sequence diagram in Figure 3 involves several interacting components within the system architecture. The ‘Monitoring Agent (MA)’ initiates the process by dispatching distributed software metrics to the ‘Metric Exchange’. Subsequently, the ‘Metric Collector (MC)’ receives these metrics from the exchange for efficient storage within the system’s database (‘Database’). Once stored, these metrics undergo continuous aggregation and analysis by the ‘Aggregator’, which provides a comprehensive view of the system’s software landscape and publishes the aggregated metrics to the metric exchange.

Simultaneously, the ‘Predictor’ comes into play by leveraging either the gathered data or the aggregated data to generate insightful software predictions. These predictions are relayed back through the ‘Metric Exchange’ to the ‘MC’ for further storage within the database. This cyclic process of data collection, aggregation, prediction, and storage enables the system to conduct real-time monitoring of software metrics and, finally, predict them.

## 5. The Dataset

To create the dataset, an execution of the proposed architecture was conducted. The MA continuously gathered metrics using parallel threads, with one dedicated thread per metric per software application. This ensured the real-time collection of data at intervals of one second. The resulting data were then stored in a database and exported as a .CSV file that was specifically tailored for the training of machine learning models to ensure compatibility and efficiency. In the .CSV file, each column corresponded to a specific metric, and each row represented a timestamped entry that captured the real-time values of these metrics.

Since the MA collected data without exceptions, missing values were nonexistent within the dataset. However, if a software application was terminated during data collection, the corresponding observations had empty values for that specific application and timeframe.

The dataset collected and used for this work was created from a whole set of software applications and their Linux processes and threads running in an operating system (OS). To list and monitor these processes, robust Linux inventory tools were leveraged. In particular, *ps* and *proc* were pivotal. The *ps* command acts as a versatile utility that offers a snapshot with detailed information on the whole set in a system. Meanwhile, the *proc* filesystem provides an interface with kernel data structures, facilitating the extraction of essential information in a human-readable format. The dataset used in this study comprised an extensive collection of software application metrics that encompassed over 500 individual processes and threads. Each process contributed a comprehensive array of metrics, yielding more than 1000 values for each of the 17 distinct metrics tracked per process. These metrics collectively captured various facets of system behaviour and resource utilisation, and they included the following:

1. Utime: The total amount of CPU time used by a process in user-space tasks.
2. Stime: The total amount of CPU time used by a process in kernel-space tasks.
3. I/O wait: The amount of time for which the CPU was idle due to waiting for I/O operations.
4. Number of threads: The total number of threads spawned by a process.
5. RSS: resident set size: the amount of memory occupied by a process in RAM.
6. VSZ: virtual set size: the total virtual memory used by a process.
7. Write bytes: The total number of bytes written by a process to the disk.
8. Read bytes: The total number of bytes read by a process from the disk.
9. CPU priority: Niceness of the value of a process.
10. Average percentage of CPU: The average percentage of CPU used by a process since its start.
11. Average percentage of memory: The average percentage of memory used by a process since its start.
12. Voluntary context switches: The number of voluntary context switches performed by a process.
13. Involuntary context switches: The number of involuntary context switches performed by a process.
14. Number of open file descriptors: The total number of file descriptors opened by a process.
15. Major faults: The number of major page faults that occurred.
16. Minor faults: The number of minor page faults that occurred.
17. Total CPU: The total CPU utilization across the entire system. This metric was gathered at the system level for its future use in aggregation.

### Correlation Matrix

To understand how the gathered metrics were correlated, a Pearson correlation matrix was created. It provided insights into the relationships between different metrics measured within the single processes that formed the software applications. It was a (K × K) square and symmetrical matrix whose *ij* entry was the correlation between the columns *i* and *j* of the metric [28]. Each value in the matrix represented the correlation coefficient between pairs of metrics. A coefficient close to 1 indicated a strong positive correlation, while a coefficient close to −1 suggested a strong negative correlation, which meant that the two variables tended to move in opposite directions. Furthermore, a value near 0 indicated that there was no relationship between them.

**Figure 4:**
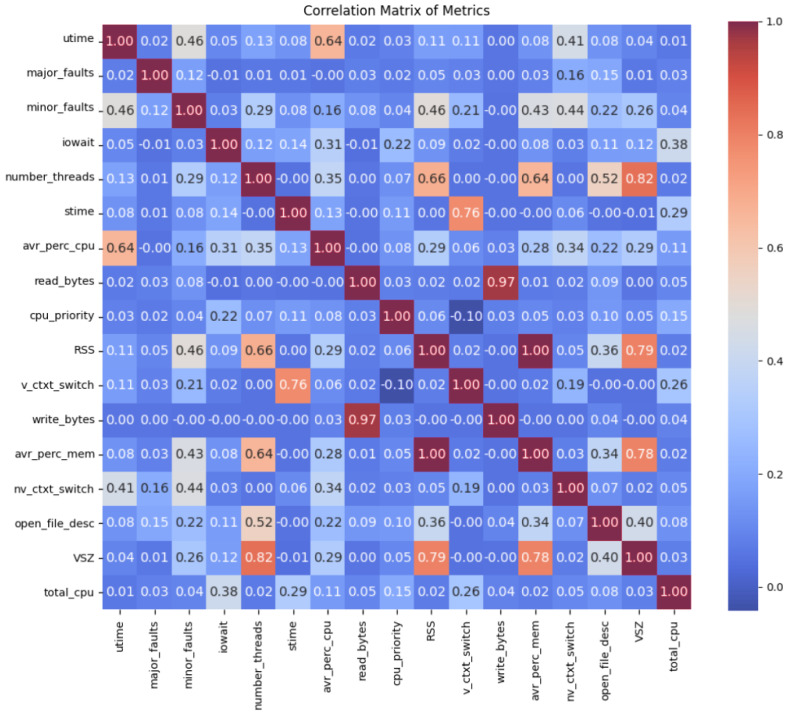
Correlation matrix showing the relationships between different metrics measured within the single processes that formed software applications.

The Pearson correlation matrix can be seen in Figure 4. For instance, the average percentage of CPU metric exhibited notably high positive correlations with utime and percentage of memory, with both of them being 0.64. This indicated that an increase in CPU usage corresponded to a greater user CPU time and memory usage. Moreover, the correlation between the number of threads and VSZ was notably strong (0.82), implying a very close relationship. This suggested that an increase in the threads of a software application could lead to greater memory utilisation. On the contrary, some metrics demonstrated opposite correlations. Write bytes and voluntary context switches displayed a very weak correlation (close to 0), suggesting that there was a minimal linear dependency between these metrics. This lack of correlation indicated that any change made in one metric did not impact the other, highlighting their independent behaviour.

It is important to note that this correlation matrix involved the calculation of relationships between variables and it does not imply causation. A strong correlation between two metrics does not necessarily indicate that a metric causes the other to change, but it means that there is a statistical relationship between them. Also, this dataset was taken from real computer operations; therefore, certain metrics may not have suffered large changes during their gathering. Specifically, the CPU priority of individual processes and the occurrence of major faults displayed limited variability within the dataset. This consistency can be attributed to intrinsic factors in typical computer usage scenarios. The CPU priority, for instance, is generally managed by the system and is not explicitly manipulated by the user under the usual working conditions. Also, unless it is under specific conditions or configurations, this metric might not change in the whole life of the software due to its dependence on system defaults or automatic adjustments. In a similar manner, the occurrence of major faults in software applications is an infrequent event during routine computer usage. In regular operation, modern computing environments are optimised to minimise such major faults, resulting in their limited occurrence.

## 6. Prediction Algorithm

The random forest algorithm was the algorithm chosen for the prediction of the metrics. It was demonstrated to be a robust learning technique in Section 2. It demonstrates prowess in handling complex nonlinear relationships within data. Each tree in the random forest aimed to minimise the total difference between the actual values of the metrics and the model prediction. Mathematically, this difference was quantified with the following formula:

$$\sum_{i=1}^{N}{\left(Y_{i}-\hat{Y_{i}}\right)}^{2}$$

where $Y_{i}$ represents the true value, and $\hat{Y_{i}}$ signifies the predicted value for each sample *i* within the training set.

**Figure 5:**
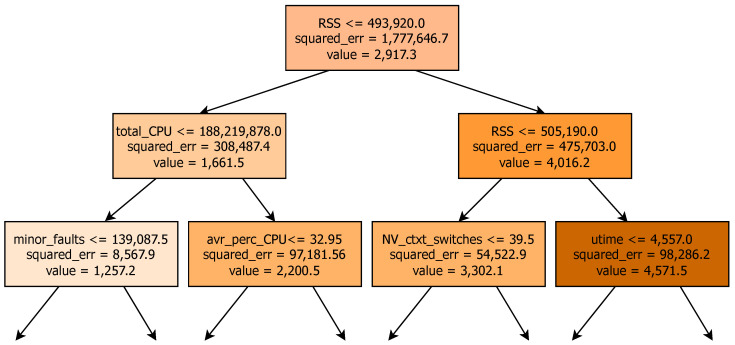
Example of a tree in the proposed model with successful results.

In a random forest model composed of numerous decision trees, each tree was made by considering a subset of features at each node. This subset was randomly chosen, allowing different trees to use different features to make decisions. The nodes in each tree represented decision points based on specific features and their associated thresholds. Figure 5 shows an example of a tree in our data with successful results, and the tree was shortened to only seven nodes to facilitate visualisation.

In each node, the feature name is seen, and the initial node indicates the feature RSS. The threshold value specifies the cutoff point for that feature. For instance, a threshold of 493,920.0 means that if the value of the RSS in a data point is greater than this threshold, the model proceeds down to the right child, where the feature RSS is found again; otherwise, it goes left, with the total CPU being used as a feature in this case.

The children (left or right) indicate if a decision moves based on the evaluation of the condition at the node. If the condition is satisfied, the model proceeds to the left child; if not, it goes to the right child. In this case, the left is the total CPU, and the right is the RSS again. Furthermore, nodes that do not have any children are considered leaf nodes, and they make the final prediction. In this example, the tree representation was restricted to a depth of three for easier visualisation. There were different trees (as many as the estimators, see Section 6), and each tree was able to split the data in different ways based on the random selection of the features. Because of this, the nodes, features, and thresholds could vary across the trees. The depth indicated the number of splits that it had; the more levels, the more complex the model. The number of nodes reflected how many decision points the tree had to make before arriving at a prediction.

In summary, the tree began by examining the specific metric that would be predicted. Then, it leveraged other features through decision points based on thresholds, as explained previously. These thresholds guided the tree along different branches, examining relationships between the rest of the metrics and the one selected for prediction. Each of the trees explored decision paths that were different from the rest. Finally, combining each of them led to the final prediction.

### The Proposed Model

The model hyperparameters were empirically chosen in order to optimise both performance and efficiency. The limit of trees—also called estimators—was set to 50. The more trees, the better robustness, but at the same time, this increased the computational effort. The model stopped splitting the nodes when it arrived at the maximum depth (in this case, it was set to 100) or if further division did not significantly improve the performance of the model. The mean squared error was chosen to measure the quality of a split (see Figure 5). There was no minimum number of leaf nodes. The random state of the data was crucial in the case of this work because we were working with time series. In order to avoid interfering with the temporal nature of the data, they were sorted by time. The input and output of the model were values that were normalised between 0 and 1. This ensured uniformity and consistency in the data, preventing features with larger scales from dominating the model’s learning process and enabling efficient convergence during training.

## 7. Empirical Results

This section details the experimental setup and findings when employing the random forest model to forecast metrics within a software application environment. The creation of the dataset and the validation results were performed on a computer with an Intel(R) Xeon(R) CPU E5-2630 v4 @2.20 GHz with 20 cores and 32 GB of RAM running Ubuntu 20.04 LTS as the operating system.

The aggregator component was used to aggregate the metrics, meaning that it listed the needed metrics that were taken from the database or created new metrics that could not be directly taken from the SO kernel. For the empirical results shown in this section, the aggregator prepared a set of the last five values of each metric. This allowed for time-series forecasting while taking the previous values of the metrics into account. An example of the creation of metrics can be the instant percentage of CPU; to calculate it, measurements of metrics at different times are needed:

(1) $$\begin{array}{cc} \mathrm{Instant}\;\mathrm{percentage}\;\mathrm{CPU} & =\frac{\left(ncpu\times 100)(\Delta utime+\Delta stime\right)}{\Delta total\;time}\; \end{array}$$

(2) $$\begin{array}{cc} & \;=No.\;of\;CPUs*\frac{\Delta (CPU\;time\;spent\;by\;the\;process)}{\Delta (CPU\;time\;spent\;by\;the\;system)}*100 \end{array}$$

Another aggregated metric was the total number of context switches (CSs), which was obtained by summing the involuntary and voluntary CSs. Write bytes per second and involuntary context switches per second were calculated by finding the difference in the measures at every second.

(3) $$\begin{array}{c} \mathrm{Total}\;\mathrm{CS}=\mathrm{Nonvoluntary}\;\mathrm{CS}+\mathrm{Voluntary}\;\mathrm{CS} \end{array}$$

(4) $$\begin{array}{c} \mathrm{Write}\;\mathrm{bytes}/s=\Delta \mathrm{write}\;\mathrm{bytes} \end{array}$$

(5) $$\begin{array}{c} \mathrm{Involuntary}\;\mathrm{CS}/s=\Delta \mathrm{Nonvoluntary}\;\;\mathrm{CS} \end{array}$$

The aggregator was not only limited to single processes; one of its strengths was that it allowed for metrics within different software applications. For example, the average of read bytes in a group of applications could be found. It was interesting to see if there was a task being carried out by a set of processes within the system to provide wider view.

### Analysis of the Results

**Figure 6:**
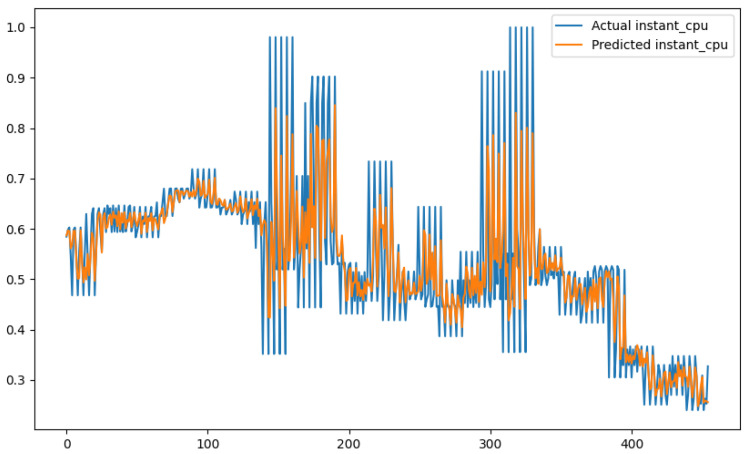
Comparison between the actual and predicted values of instant CPU usage for software application SA1.

**Figure 7:**
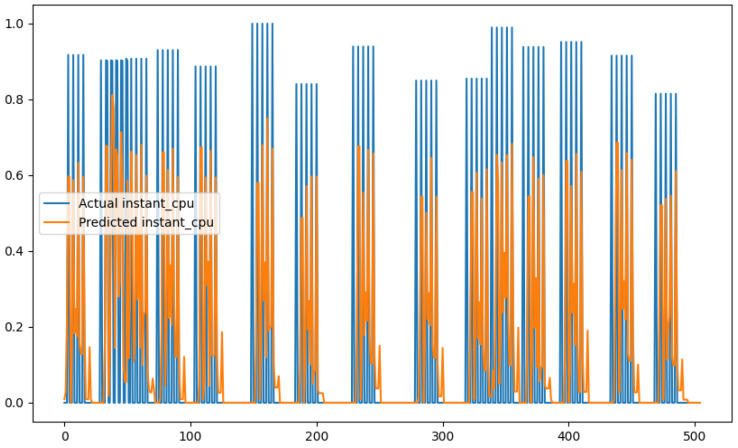
Comparison between the actual and predicted values of instant CPU usage for software application SA2.

**Figure 8:**
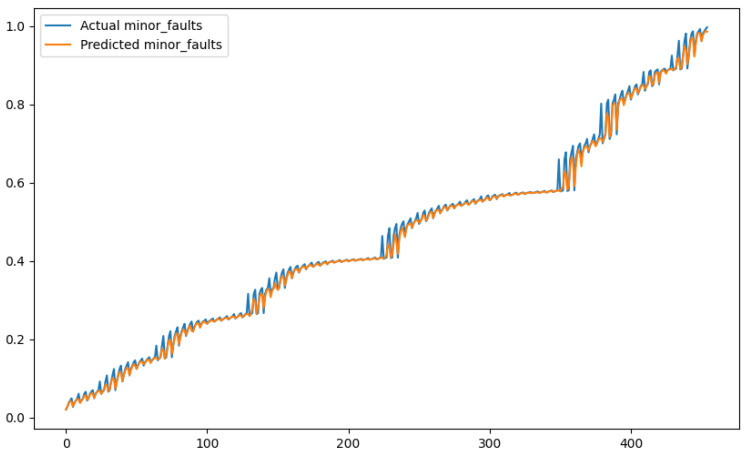
Comparison between the actual and predicted values of minor faults for software application SA1.

**Figure 9:**
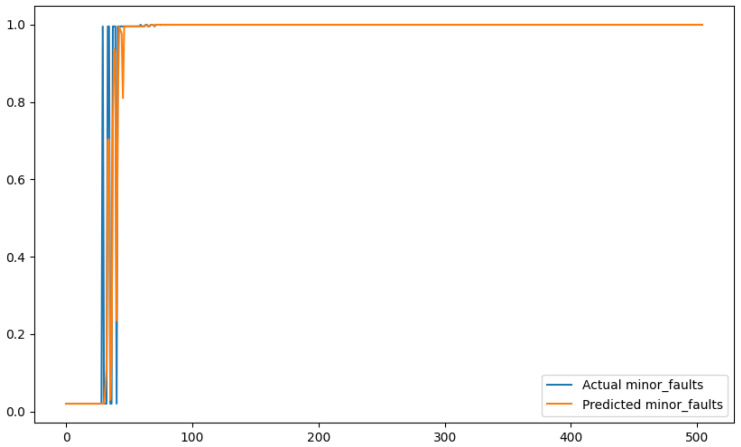
Comparison between the actual and predicted values of minor faults for software application SA2.

**Figure 10:**
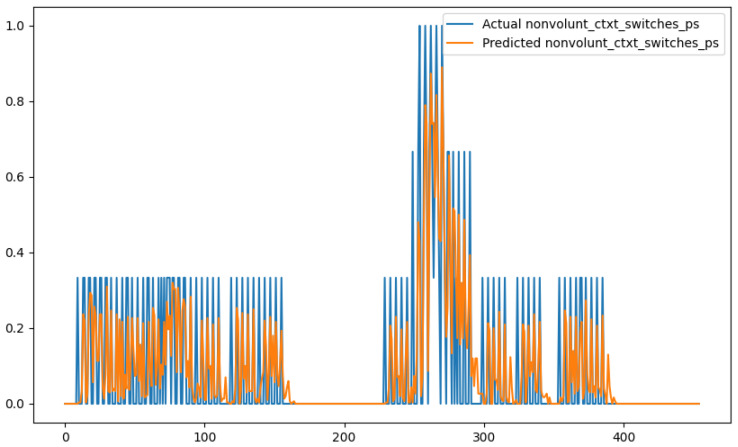
Comparison between the actual and predicted values of involuntary context switches for software application SA1.

**Figure 11:**
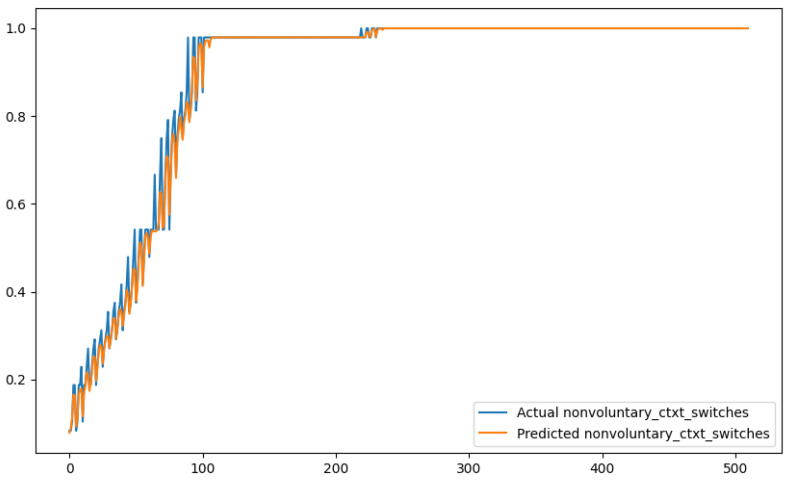
Comparison between the actual and predicted values of involuntary context switches for software application SA2.

**Figure 12:**
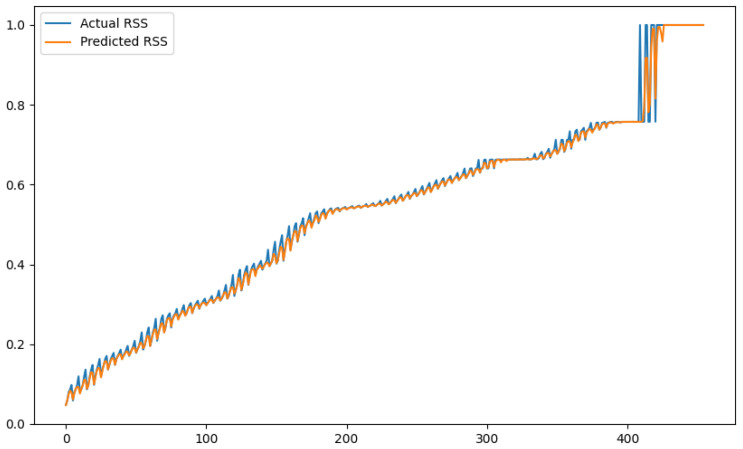
Comparison between the actual and predicted values of RSS for software application SA1.

**Figure 13:**
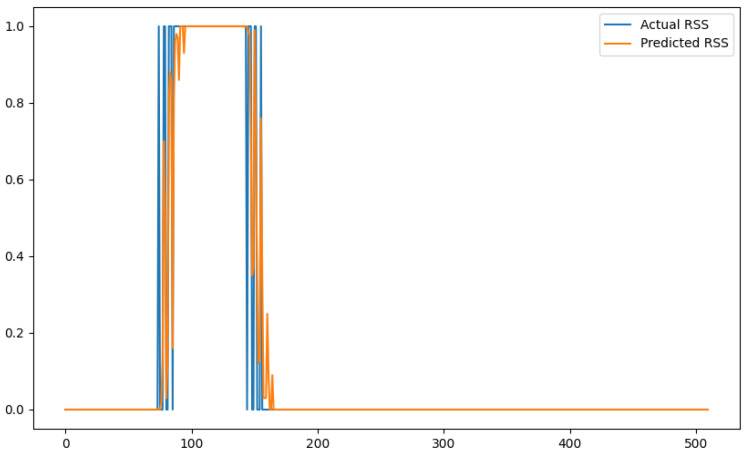
Comparison between the actual and predicted values of RSS for software application SA2.

**Figure 14:**
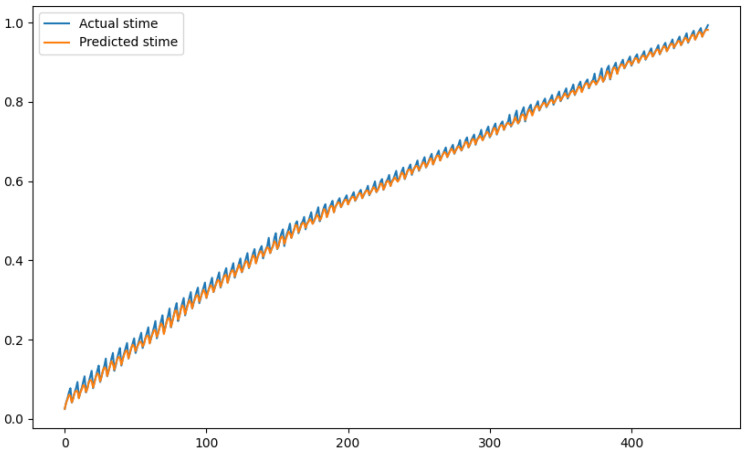
Comparison between the actual and predicted values of stime for software application SA1.

**Figure 15:**
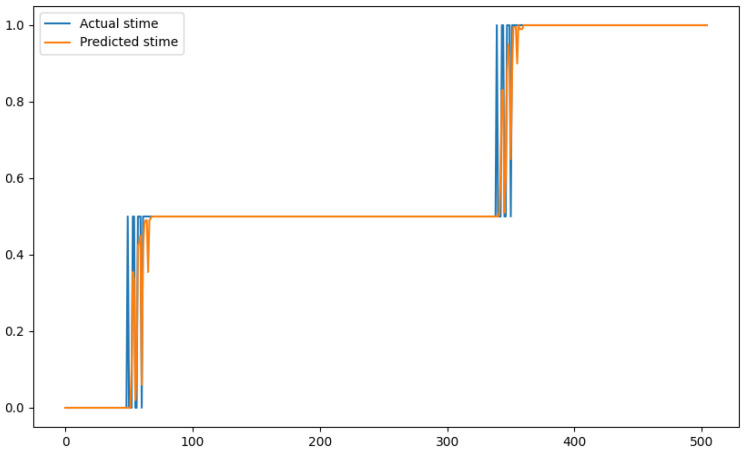
Comparison between the actual and predicted values of stime for software application SA2.

**Figure 16:**
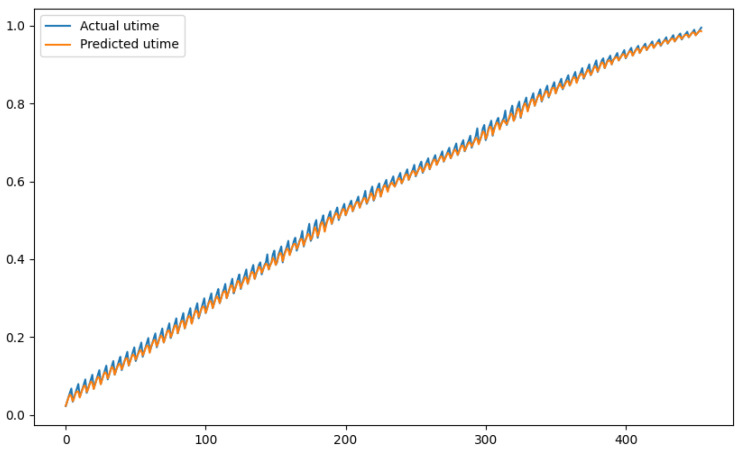
Comparison between the actual and predicted values of utime for software application SA1.

**Figure 17:**
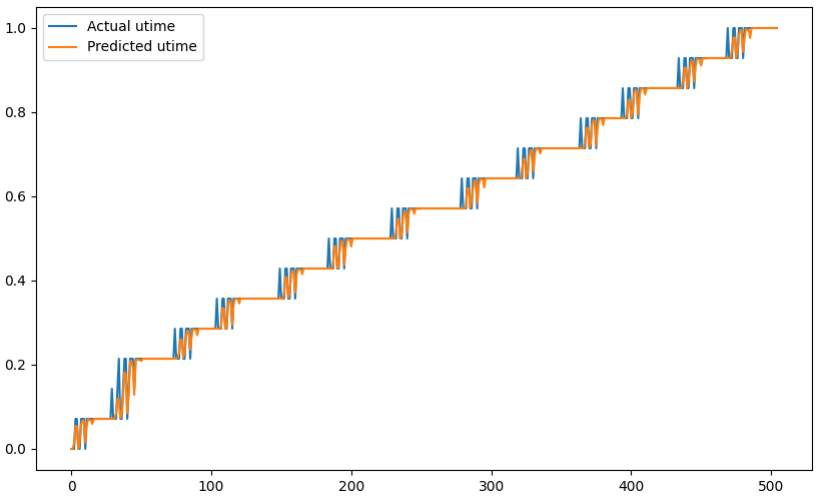
Comparison between the actual and predicted values of utime for software application SA2.

In order to show a visual representation of the forecasting results, in this section, different figures of two different software applications named SA1 and SA2 are presented. The forecasting of the instant CPU (aggregated rule calculated via Formula (1)) is seen in Figure 6 and Figure 7, where one can observe that the predicted values were usually not as high or low as the actual value, which is why the average R2 score was 0.753. Forecasting could be used to see the trend of the metric, but the exact value was something that needed to be improved. The forecasting of minor faults is seen in Figure 8 and Figure 9. Moreover, in Figure 10 and Figure 11, the representation of involuntary context switches can be observed. In Figure 12 and Figure 13, the values of the RSS are depicted. Finally, the forecasting of the total amount of CPU time used by a software application in both the user space and kernel space can be seen in Figure 14 and Figure 15 and in Figure 16 and Figure 17, respectively.

From these figures, it can be affirmed that the forecasting successfully achieved the intended behaviour. Constant or small changes in the current values were perfectly forecasted. When a large change was made, the first forecast may have lacked correctness, but a good rate of success was achieved overall.

In Table 2, a study of the results is shown. The table is divided into three main columns: R2 score, normalised mean squared error (NMSE), and average time spent for prediction.

1. $R^{2}$ (Coefficient of Determination):
  $$R^{2}=1-\frac{\sum_{i=1}^{n}{\left(y_{i}-{\hat{y}}_{i}\right)}^{2}}{\sum_{i=1}^{n}{\left(y_{i}-\bar{y}\right)}^{2}}$$
2. Mean Squared Error (MSE):
  $$\mathrm{MSE}=\frac{1}{n}\sum_{i=1}^{n}{\left(y_{i}-{\hat{y}}_{i}\right)}^{2}$$
  where:
  - $y_{i}$ = Actual value at index *i*;
  - ${\hat{y}}_{i}$ = Predicted value at index *i*;
  - $\bar{y}$ = Mean of the actual values;
  - *n* = Number of samples.

As the data used for both training and predicting were normalised between 0 and 1, this contributed to a normalised evaluation of the different scales found in the features. Because of this, the MSE was defined as the NMSE in Table 2. In summary, these equations are fundamental in evaluating the performance of regression models. $R^{2}$ and the MSE quantify the goodness of fit and accuracy, respectively.

Regarding the results, in general, minimal variation was observed. When the metrics remained constant or exhibited minimal changes, the random forest was able to predict them with an impressively low error. These cases could be seen in numerous software applications that did not intensively consume resources, which contributed to the model’s achievement of good results overall. An example could be the metric with the best results, CPU priority (0.980 R2 score and 0.0005 NMSE). This metric did not suffer major changes during normal software application use; therefore, it largely did not change. Furthermore, the total CPU showed the second best R2 score (0.959); it is an important metric due to its implications for the calculation of the instantaneous CPU usage (see examples of the aggregated rule in Figure 6 and Figure 7). While most of the metrics displayed accurate predictions, it is worth noting that the normalisation process adjusted these metrics, which could potentially reach unscaled values of up to 1 $\times \;10^{6}$, thereby influencing the observed NMSE values. For instance, while an NMSE of 0.0016 might suggest near-perfect predictions, especially for metrics with larger scales, such as ‘write bytes,’ a discrepancy of 100 in a prediction within a scale of 1e6 can lead to a relatively low error score. This emphasises the significance of scale in interpreting error metrics for regressors such as the random forest model. In essence, while the model demonstrated robust predictive abilities, especially in stable or minimally changing metrics, understanding the context of scaling is essential for a more nuanced interpretation of error metrics such as the NMSE and R2 score.

The delay between metric predictions was determined by the MA. In essence, when the MA monitored metrics every second, the predictor forecasted for the upcoming second. It is noteworthy that the delay is fully configurable and can be changed in order to meet the requirements depending on the use case. Furthermore, the model was able to generate forecasts for more than five of the seventeen metrics per second with a speed that was consistently below 0.16 s per metric.

This can provide real-time metric gathering and forecasting in industrial computing scenarios. The distributed architecture allowed us to gather and predict metrics from different computers and software applications at the same time. The inherent scalability of the architecture ensures its effectiveness even in complex setups with numerous running software applications. As the system dynamically adapts to increases in the number of processes or threads created by multiple software applications, it maintains high levels of performance in terms of metric gathering and forecasting. It is also important to understand that the more software applications are running, the more processes or threads will be created, and the higher load on the architecture, as it will need to gather all of them and then predict what the aggregator provides.

## 8. Discussion

Our chosen data collection focused on normal operation scenarios. While it provided the closest representation of real-world conditions, it is true that some software applications in a distributed environment may exhibit new and atypical behaviours due to unexpected events or failures. The parameter prediction within our current work focused on accurately forecasting application metrics for resource management and optimisation. Furthermore, we acknowledge that identifying and anticipating potential failures requires additional analyses and decision-making capabilities. An expansion of our approach by incorporating a dedicated “failure detector” agent that leverages the predicted metrics to trigger alerts and initiate appropriate countermeasures in response to identified anomalies or unusual parameter deviations is envisioned as future work. This proposed improvement will further enhance the overall resilience and proactive fault tolerance in industrial scenarios. Moreover, a future step forward would be to create a controlled scenario in which some software applications may change their behaviour unexpectedly. Examples include preparing a threat to an application, forcing the application to carry out determined behaviours, or programming a new software application to perform a specific task. In addition, the metrics chosen in this study mainly included the software application CPU, memory, and faults. These metrics offer valuable insights into a process’s behaviour and resource utilisation. However, incorporating network-related metrics could allow deeper visibility, but accessing these network metrics though the OS kernel might entail significant time and resource consumption, potentially limiting the real-time capabilities that we need to achieve.

In Section 2, deep research was conducted in order to justify the election of the algorithm and its success rate compared to those of other prediction models. However, the study suggested that future research could examine further model implementations, such as linear regression or deep learning, and how they can improve aggregated metrics, which are the metrics for which the worst results were seen. However, in this research, the main goal was to provide an AI-enabled architecture that was able to sense and predict software applications rather than a review of algorithms.

Additionally, in the realm of orchestrating software components within the landscapes of Industry 4.0 and 5.0, the integration of tools such as Kubernetes [29] or OpenShift [30] is becoming more common. These tools not only facilitate efficient deployment and scaling but also play a key role in optimising the overall operational efficiency of distributed systems.

As a result of the realisation of this work, further promising research can be suggested. It was noticed that there is an important gap in the literature regarding software applications in this new era of the change from manual to virtual industry. This study introduced a baseline for the comprehension and prediction of software applications—from the sensing of process and threads that compose the whole system until actions depending on the specific goal—which is pivotal for setting the stage for future research endeavours. For instance, this work serves as a starting point for potential advancements such as the development and integration of a novel decision-making component that is explicitly tailored to the enhancement of the predictive capabilities and operational efficiency of software applications within this evolving landscape.

## 9. Conclusions

In this work, we presented and completed the creation of a new architecture that effectively gathered the metrics of software applications for the era of Industry 4.0 and 5.0. With this architecture, we created a dataset with information on 17 metrics that were taken directly from the OS kernel. Moreover, an AI algorithm called “random forest” was implemented as a final state in order to achieve real-time metric forecasting. This whole architecture allowed us to not only sense the system performance by understanding each of the running processes, but also to forecast software behaviour. The results of the forecasting for the whole set of metrics were good overall, with an R2 accuracy that was usually greater than 0.9. Some metrics, such as the instant CPU percentage, write bytes per second, and involuntary context switches per second, have to be further analysed. Regarding the speed of the approach, it lasted less than 0.16 s per metric, thus providing the forecasting of up to five metrics per second in the implemented prototype system. Furthermore, a comparison between the forecasted values and current values was depicted for a better visualisation. In addition, the whole set of errors (R2 score and NMSE) and the average time spent for each metric were shown to demonstrate the satisfactory results of this approach. Finally, we discussed the findings and their implications, as well as potential future work.

## Figures and Tables

**Table 1:** Comparison of research works.

Criteria	[24]	[25]	[21]	[19]	[26]	[22]	[23]	[18]	This Study
Dataset creation	✘	✔	✔	✘	✔	✔	✔	✘	✔
Number input metrics	7	33	6	1	4	+1 *	6	17	17
Number output metrics	1	1	2	1	1	1	1	1	17
RT metric gathering	✔	✘	✔	✔	✘	✘	✔	✘	✔
Automated architecture	✔	✘	✔	✔	✘	✘	✔	✘	✔
Distributed architecture	✘	✘	✘	✔	✘	✘	✘	✘	✔
RT prediction	✔	✘	✔	✔	✘	✘	✔	✘	✔
Validation ^#^	(S)	(A)	(S)	(P)	(A)	(A)	(A)	(A)	(P)

(#) Validation: analytical (A), simulation (S), prototype (P); (*): not specified in the paper.

**Table 2:** R2 score, NMSE, and average time for the prediction of metrics.

Metrics	R2 Score	NMSE	Average Time (s)
utime	0.937	0.0067	0.12
major faults	0.944	0.0056	0.13
minor faults	0.908	0.0115	0.14
iowait	0.926	0.0081	0.15
number of threads	0.928	0.0092	0.16
stime	0.922	0.0016	0.11
average perc CPU	0.948	0.0011	0.10
read bytes	0.946	0.0011	0.12
CPU priority	0.980	0.0005	0.10
RSS	0.913	0.0104	0.13
voluntary ctxt switches	0.927	0.0031	0.14
write bytes	0.912	0.0116	0.15
average perc mem	0.915	0.0041	0.11
involuntary ctxt switches	0.947	0.0022	0.11
number open file descriptor	0.920	0.0115	0.13
VSZ	0.955	0.0031	0.10
total CPU	0.959	0.0021	0.14
instant perc CPU	0.753	0.0207	0.16
write bytes/s	0.587	0.0457	0.15
involunt ctxt switches/s	0.806	0.0071	0.11
total ctxt switches	0.941	0.0031	0.13

## Data Availability

The dataset presented in this article is not readily available because the data is part of an ongoing study.

## Abbreviations

The following abbreviations are used in this manuscript:

OS	Operating System
AI	Artificial Intelligence
MA	Monitoring Agent
CS	Context Switch
CPU	Central Processing Unit
IO	Input/Output
RSS	Resident Set Size
VSZ	Virtual Memory Size
SA	Software Application
RT	Real Time
RF	Random Forest
ARIMA	Autoregressive Integrated Moving Average
KPI	Key Performance Indicator
